# Identification of Human Ovarian Adenocarcinoma Cells with Cisplatin-Resistance by Feature Extraction of Gray Level Co-Occurrence Matrix Using Optical Images

**DOI:** 10.3390/diagnostics10060389

**Published:** 2020-06-09

**Authors:** Chih-Ling Huang, Meng-Jia Lian, Yi-Hsuan Wu, Wei-Ming Chen, Wen-Tai Chiu

**Affiliations:** 1 Center for Fundamental Science, Kaohsiung Medical University, Kaohsiung 807, Taiwan; 2 School of Dentistry, College of Dental Medicine, Kaohsiung Medical University, Kaohsiung 807, Taiwan; sy2es93103@gmail.com (M.-J.L.); bill321cm1@gmail.com (W.-M.C.); 3 Department of Medicinal and Applied Chemistry, College of Life Science, Kaohsiung Medical University, Kaohsiung 807, Taiwan; qoo860724@gmail.com; 4 Department of Biomedical Engineering, National Cheng Kung University, Tainan 701, Taiwan; wtchiu@mail.ncku.edu.tw

**Keywords:** chemoresistance, cisplatin, gray-level co-occurrence matrix, ovarian adenocarcinoma

## Abstract

Ovarian cancer is the most malignant of all gynecological cancers. A challenge that deteriorates with ovarian adenocarcinoma in neoplastic disease patients has been associated with the chemoresistance of cancer cells. Cisplatin (CP) belongs to the first-line chemotherapeutic agents and it would be beneficial to identify chemoresistance for ovarian adenocarcinoma cells, especially CP-resistance. Gray level co-occurrence matrix (GLCM) was characterized imaging from a numeric matrix and find its texture features. Serous type (OVCAR-4 and A2780), and clear cell type (IGROV1) ovarian carcinoma cell lines with CP-resistance were used to demonstrate GLCM texture feature extraction of images. Cells were cultured with cell density of 6 × 10^5^ in a glass-bottom dish to form a uniform coverage of the glass slide to get the optical images by microscope and DVC camera. CP-resistant cells included OVCAR-4, A2780 and IGROV and had the higher contrast and entropy, lower energy, and homogeneity. Signal to noise ratio was used to evaluate the degree for chemoresistance of cell images based on GLCM texture feature extraction. The difference between wile type and CP-resistant cells was statistically significant in every case (*p* < 0.001). It is a promising model to achieve a rapid method with a more reliable diagnostic performance for identification of ovarian adenocarcinoma cells with CP-resistance by feature extraction of GLCM in vitro or ex vivo.

## 1. Introduction

Ovarian cancer is the most malignant of all gynecological cancers [1]. A challenge that deteriorates with ovarian adenocarcinoma in neoplastic disease patients has been associated with the chemoresistance of cancer cells. Cisplatin (CP) is a platinum-containing compound, which belongs to the first-line chemotherapeutic agents for the treatment of human ovarian cancer [2]. Therefore, it would be beneficial for cancer therapy to identify various chemoresistance for human ovarian adenocarcinoma cells, especially CP-resistance.

Ovarian carcinomas consist of at least five distinct diseases: high-grade serous, low-grade serous, clear cell, endometrioid, and mucinous [3]. High-grade serous ovarian cancer is responsible for approximately 80% of ovarian cancer cases and two-thirds of ovarian cancer deaths. OVCAR-4 ranked as one of the highest matches to high-grade serous ovarian cancer, a cell-line collected from a 42-year-old ovarian cancer patient and found to be resistant to combination chemotherapy [4]. A2780 is serous carcinoma cell line with platinum sensitivity [5]. IGROV1 is human clear cell type ovarian carcinoma cell line. It has the propensity to float as clusters isolated from tumor tissue and ascites [6].

Gray level co-occurrence matrix (GLCM) characterizes the texture of images by calculating from a numeric matrix [7], which was defined by Haralick et al. [8]. It can be used to analyze the medical imaging from magnetic resonance imaging or ultrasonography and find the potential relationship with tumor malignancies [9], such as brain tumor detection [10], liver tumors [11], and histopathological images [12].

In general, it is not easy to take images of ovarian cancer, but ovarian cancer cells can be extracted from ascites of patients. The cells can be cultured mono-layered to dissolve the problem for the image taken. In our previous study, detection of various characteristics of cancer cells by feature extraction of GLCM can be applied in real clinical cases for metastatic cancer cells [13] or biopsy [14] images which were successfully taken from camera.

In search of novel mechanisms that may lead to CP chemoresistance, scientists used a lot of cells and subtractive hybridization to identify differentially expressed genes [15]. However, chemoresistance happens in cells where equivalent effects of various expressed genes is expressed outside of cell imaging. In this study, we proposed a promising method based on GLCM image processing model to achieve a rapid method with a more reliable diagnostic performance for various chemoresistance for CP of human ovarian adenocarcinoma cells by feature extraction of GLCM.

## 2. Materials and Methods

The optical image system used in this study comprised a microscope (BX-53 OLYMPUS, Tokyo, Japan) and DVC camera (Model: 1500M-T1-GE S/N 3797) with an image capture software (DVC View™). The different images (1392 × 1040 pixels) of the samples were obtained. The diameter of a cell was approximately 10–20 pixels in obtained images and the processing image was 150 × 150 pixels with 256 gray level for extracting the characteristic texture feature of cells [13].

GLCM was used to calculate contrast, energy, entropy, and homogeneity to analyze the images texture features. The variable C (*i*, *j*) expressed in Equations (1)–(4) refer to the value at the (*i*, *j*) position in a GLCM. These four indexes corresponded to the disorder in cell images and indicate the surface characteristic of cancer cells. For example, contrast displayed the edges and three-dimensional (3D) structures of cell; energy represented the orderliness; homogeneity represented the smoothness of the distribution for gray level and entropy showed the disorder degree.

(1) $$\mathrm{Contrast}:\;\sum_{ij=1}^{G}C_{ij}{\left(i-j\right)}^{2}$$

(2) $$\mathrm{Energy}:\;\sum_{ij=1}^{G}C_{ij}{}^{2}$$

(3) $$\mathrm{Homogeneity}:\;\sum_{ij=1}^{G}\frac{1}{1+\left|i-j\right|}C_{ij}$$

(4) $$\mathrm{Entropy}:-\sum_{ij=1}^{G}C_{ij}\log C_{ij}$$

Due to ovarian cancer being the most malignant of all gynecological cancers its chemoresistance was challenging the first-line chemotherapeutic agents for the treatments. High-grade serous ovarian cancer is responsible for approximately 80% of ovarian cancer cases so we selected human serous type ovarian adenocarcinoma cell lines (OVCAR-4 and A2780), and human clear cell type ovarian carcinoma cell line (IGROV1) to demonstrate the GLCM texture feature extraction and analysis of images. Furthermore, wild type (WT) human ovarian adenocarcinoma cell lines and that with chemoresistance for CP were used. All cells were maintained in RPMI1640 medium solution (Gibco) containing 10% fetal calf serum and incubated at 37 °C with 5% CO_2_. A2780 were cultured in PMI1640 medium with non-essential amino acids, glutamine, and 0.5 units insulin.

Before acquiring the images, cells were cultured with cell density of 6 × 10^5^ in a glass-bottom dish. A silicone separator (Culture-Insert 2 Well, iBidi, Martinsried, Germany) was placed to trap cells to form a uniform coverage of the glass slide and create a clear region as the blank. Samples were incubated for 48 h and then washed twice in a phosphate buffered saline solution (PBS, 0.1 M, pH = 7.4).

To evaluate the reliability of the detection method, the statistical differences were evaluated using a one-way analysis of variance (ANOVA) technique. In evaluating the test results, a * *p* value of <0.05 was statistically significant, a ** *p* value of <0.01 was very statistically significant, and a *** *p* value of <0.001 was highly statistically significant.

## 3. Results and Discussion

**Figure 1:**
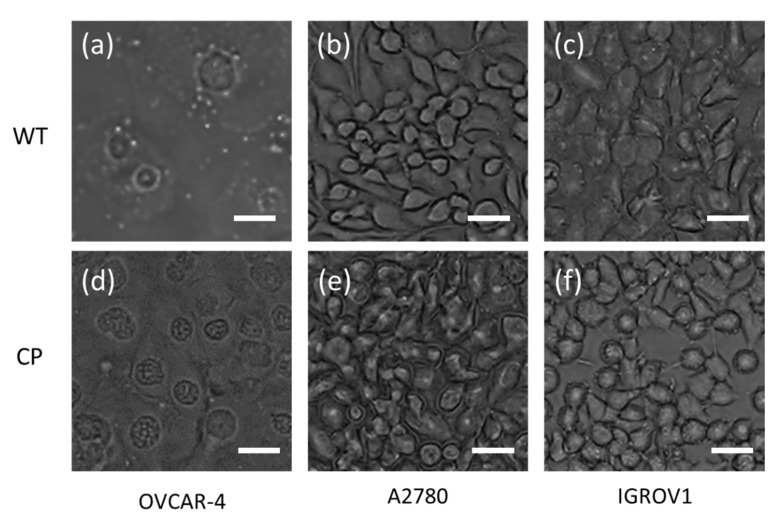
Cell images for serous cell type (**a**) OVCAR-4, (**b**) A2780, and (**c**) clear cell type IGROV1 of wild type (WT) ovarian adenocarcinoma cells and (**d**–**f**) which were cisplatin-resistant (CP). (Scale bar: 20 μm)

Figure 1 shows cell images for serous cell type of OVCAR-4, A2780, and clear cell type IGROV1 of WT and CP-resistantance of ovarian adenocarcinoma cells. Under the optical microscope, little morphological differences could be observed between the WT ovarian adenocarcinoma cells (Figure 1a–c) and its CP-resistant counterpart (Figure 1d–f). However, chemoresistance is a very complex phenomenon, and it involves multiple interconnected mechanisms [16]. CP is localizing to the nucleus and binding to DNA, and then it gives rise to intrastrain DNA adducts. Subsequently, cancer cells apoptosis was caused by triggering G2 cell cycle arrest [17]. In a previous study, ovarian adenocarcinoma cells with CP-resistance recovered a normal proliferation state after a treatment with 5 μg/mL CP for 41 days. At confluence, the cell layer displayed some morphological differences and cells were becoming able to pile and to form three dimensional spherical structures [18]. It means that the cells with chemoresistance were tending to form stereoscopic structures and this characteristic can be analyzed by GLCM texture feature extracting of images. It will be promising and potentially detect chemoresistance before the new therapeutics.

**Figure 2:**
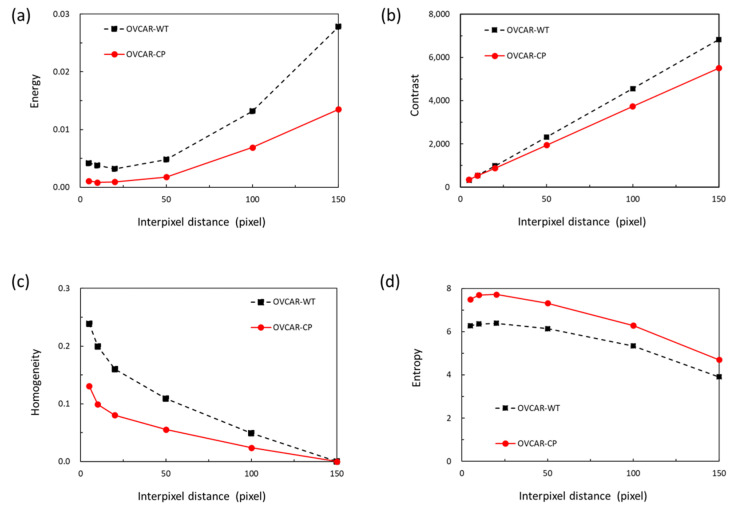
Gray level co-occurrence matrix (GLCM) texture feature: (**a**) energy, (**b**) contrast, (**c**) homogeneity, and (**d**) entropy for serous type (OVCAR-4) of ovarian adenocarcinoma cells with various interpixel distance with wild type (WT) and chemoresistance for cisplatin (CP).

Figure 2 shows GLCM texture features of energy, contrast, homogeneity, and entropy for serous type (OVCAR-4) of ovarian adenocarcinoma cells with various interpixel distance with chemoresistance for CP and WT cells. The inflection points of texture feature can be found in Figure 2 around interpixel distances of 10–20 pixels. The size of the image used for processing is 150 × 150 pixels and the diameter of ovarian adenocarcinoma cells is approximately 10–20 pixels, which implies that the characteristic texture feature commonly occurs in the boundary of cells, and that we can set up the interpixel distance as a specific value (e.g., 10 pixels) around the cell diameter to obtain the typical texture features. The ovarian adenocarcinoma cells with CP-resistance exhibit more 3D structures with characteristic rough surfaces. These structures enhance the optical scattering effect and it is difficult to observe the cells in the same focus plane and enhance the margins of cells. These characteristics indicate that the images of ovarian adenocarcinoma cells with CP-resistance have lower energy and homogeneity but higher contrast and entropy due to the morphologies. These four texture features can be used to predict the ability for CP-resistance of ovarian adenocarcinoma cells.

**Figure 3:**
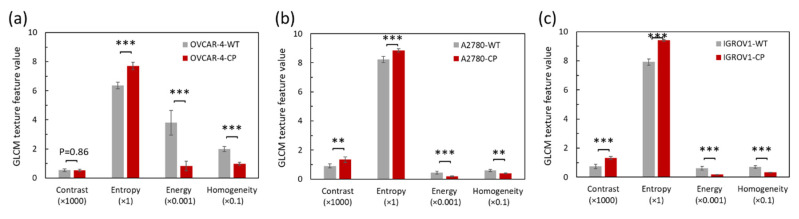
Gray level co-occurrence matrix (GLCM) texture feature of wild type (WT) and cisplatin (CP)-resistant ovarian adenocarcinoma cells for (**a**) OVCAR-4, (**b**) A2780, and (**c**) IGROV1.

Texture feature of GLCM extracting for WT and CP-resistant ovarian adenocarcinoma cell images are shown in Table 1. The texture features of WT ovarian adenocarcinoma cells were used as the benchmark and statistically compared with those of the CP-resistant cells for OVCAR-4, A2780, and IGROV-1, respectively. In general, the results show that for each of the cell lines, the CP-resistant ovarian adenocarcinoma cells have a higher contrast and entropy than the WT. Figure 3 shows GLCM texture features of WT and CP-resistant ovarian adenocarcinoma cells for OVCAR-4, A2780, and IGROV1. Table 1 and Figure 3 show that almost the differences in these four GLCM texture features of CP-resistant and WT cells were statistically significant in every case (*p* < 0.01 or *p* < 0.001). In other words, it provides the means to reliably differentiate between WT and CP-resistant cells for all three cell lines.

Due to the multi-factor analysis being complex, signal-to-noise (S/N) ratios of these four GLCM texture features for WT and CP-resistant ovarian adenocarcinoma cells were calculated and shown in Table 2. The basic concept was according to Taguchi method [19]. In this study, S/N ratio was meaning the degree of the images influenced by the various factors. It was calculated by equations from the Taguchi method. Taguchi method was used to improve the qualities of products efficiently with parameters designed in engineering fields. S/N ratio was meaning the degree of the product influenced by the various factors. In this study, the various GLCM texture features can be the parameter of cell images. For CP-resistant type cells, two of the four GLCM texture features were for smaller-is-better (i.e., energy and homogeneity) and two for larger-is-better (i.e., contrast and entropy). It was same as the basic concept of Taguchi method, so we used it for multi-factor calculation. S/N ratio was calculated by Equations (5) and (6) [20]:

(5) $$\text{Smaller-is-better}:\;S/N\;\mathrm{ratio}=-10\log(\frac{1}{n}\sum y_{i}^{2})$$

(6) $$\text{Larger-is-better}:\;S/N\;\mathrm{ratio}=-10\log(\frac{1}{n}\sum \frac{1}{y_{i}^{2}})$$

S/N ratio can be referred to the degree for chemoresistance of cells based on GLCM texture feature extraction. The higher S/N ratio means the cell images were more like CP-resistant type. In Table 2, the S/N ratio of WT of ovarian adenocarcinoma cells were 17.93 ± 0.59, 23.37 ± 0.42, and 22.81 ± 0.43 for OVCAR-4, A2780 and IGROV1, respectively. The S/N ratio of CP-resistant ovarian adenocarcinoma cells were 21.76 ± 0.50, 24.44 ± 0.17, and 25.09 ± 0.07 for OVCAR-4, A2780 and IGROV1, respectively. The differences in these cells were statistically significant in every case (*p* < 0.001). Compared to Table 1, the results of false positive CP were eliminated using multi-factor calculation. In this study, S/N ratio was meaning the degree of the images influenced by the various factors. Moreover, multi-factors included the tumor heterogeneity caused by different cell types in vivo.

The main factors which limit treatment efficiency are recurrence and progressive acquisition of chemoresistance. Chemoresistance is a complex process involving many stages. As a result, there is an urgent requirement for low-cost, high-throughput methods for assessing the risk of chemoresistance in a timely and quantitative manner. Accordingly, this study has proposed an optical method for chemoresistance detection based on GLCM texture features extraction. The feasibility of the proposed approach has been demonstrated using three pairs of ovarian adenocarcinoma cells, namely OVCAR-4, A2780, and IGROV1. Notably, the proposed method enables physical characterization of ovarian adenocarcinoma cells even in the case where the size and morphologies of the CP-resistant cells are very similar to those of WT.

Optical images could be used to study biophysical processes in living systems and to monitor morphological and physiological changes such as precancerous or cancerous conditions [21]. In a previous study, it has been indicated that epithelial-mesenchymal transition (EMT) contributes to chemoresistance acquisition [22] and indeed CP-resistant cell images result in the formation of dense 3D structures with characteristic rough surfaces compared to wild type cells. These cell structures enhance the higher contrast and entropy, and lower energy and homogeneity in GLCM texture feature of images.

**Figure 4:**
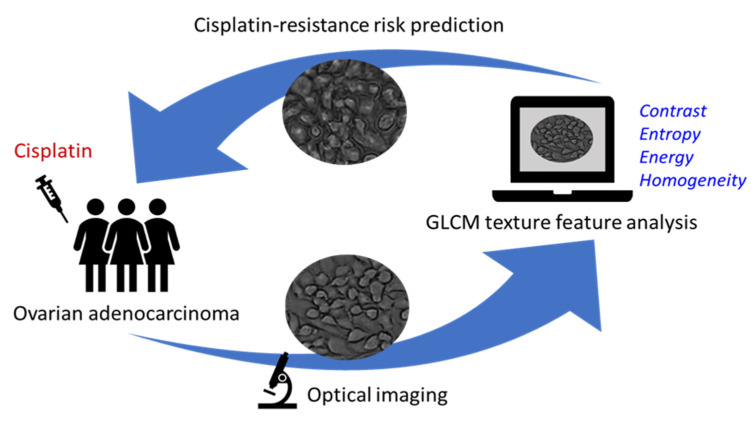
Sketch of the proposed promising model to achieve a rapid method with a more reliable diagnostic performance for identification of ovarian adenocarcinoma cells with cisplatin-resistance by feature extraction of GLCM in vitro or ex vivo.

The proposed method in this study has many advantages over in vitro tests, including a faster optical detection, a lower cost, a larger sample size, and a greater throughput. Most importantly, it provides the means to obtain a quantitative evaluation of the chemoresistance risk and therefore reduces the reliance on the practical skill and experience of the practitioner. Figure 4 shows the sketch of this promising model to achieve a rapid method with a more reliable diagnostic performance for identification of ovarian adenocarcinoma cells with cisplatin-resistance by feature extraction of GLCM in vitro or ex vivo. In the same time, machine learning was rapidly developing as the artificial intelligence technique [23]. The proposed method in this study was based on computer aided diagnosis [24] and it can be combined with the support vector machine [25] to train the model for proceeding the mass identification of chemoresistance risk for cancer cells. According to our previous study [13,14], more than ten cancer species were selected for feature extraction using optical images and we get positive results for various identification. It has potential for other cancer cells due to their equivalent effect for epithelial-mesenchymal transition expressed in cell imaging. However, it was required for the high throughput platform and combined with machine learning to train the model for proceeding the mass identification of chemoresistance risk for more cancer species. It provides a highly promising solution for physical characterization of ovarian adenocarcinoma cells with chemoresistance in vitro. Even for clinical practice, multiple invasive biopsies also can be used to analyze for feature extraction using optical images, but it was required to use scanned laser pico-projection system (SLPP) which has the narrower bandwidth compared to traditional white light to enhance the contrast and entropy of images for analysis [14].

## 4. Conclusions

Serous type ovarian adenocarcinoma cells with chemoresistance have more obvious edges and 3D structures, and the images were respected to have the higher contrast and entropy, lower energy and homogeneity. This provides the means to obtain a quantitative evaluation of the chemoresistance risk. It is a promising model to achieve a rapid method with a more reliable diagnostic performance for identification of ovarian adenocarcinoma cells with cisplatin-resistance by feature extraction of GLCM in vitro or ex vivo. In the future, the cells of the same patient could be taken from various stages of treatments to monitor morphological and physiological changes for cancerous conditions. It provides a quantitative evaluation of chemoresistance risk for chemotherapeutic agents in the next treatments. It can help to detect chemoresistance of cancer cells before the new therapeutics.

## Figures and Tables

**Table 1:** Gray level co-occurrence matrix (GLCM) texture features for wild type (WT) and cisplatin-resistant (CP) of ovarian adenocarcinoma cells.

Cell	Feature	WT	CP	*p*-Value
OVCAR-4	Contrast (×10^3^)	0.54 ± 0.08	0.53 ± 0.07	0.86
	Entropy (×10^0^)	6.36 ± 0.22	7.70 ± 0.26	***
	Energy (×10^−3^)	3.80 ± 0.84	0.83 ± 0.32	***
	Homogeneity (×10^−1^)	1.99 ± 0.18	0.99 ± 0.10	***
A2780	Contrast (×10^3^)	0.91 ± 0.13	1.35 ± 0.19	**
	Entropy (×10^0^)	8.23 ± 0.21	8.85 ± 0.11	***
	Energy (×10^−3^)	0.45 ± 0.11	0.20 ± 0.03	***
	Homogeneity (×10^−1^)	0.60 ± 0.08	0.40 ± 0.03	**
IGROV1	Contrast (×10^3^)	0.74 ± 0.15	1.32 ± 0.09	***
	Entropy (×10^0^)	7.92 ± 0.21	9.41 ± 0.06	***
	Energy (×10^−3^)	0.61 ± 0.12	0.18 ± 0.01	***
	Homogeneity (×10^−1^)	0.71 ± 0.09	0.33 ± 0.01	***

Note: GLCM sampling offset was (10.0) and compare to WT and * *p* < 0.05, ** *p* < 0.01, and *** *p* < 0.001.

**Table 2:** Signal-to-noise (S/N) ratio of gray level co-occurrence matrix (GLCM) texture feature for wild type (WT) and cisplatin-resistant (CP) ovarian adenocarcinoma cells.

Cells	WT	CP	*p*-Value
OVCAR-4	17.93 ± 0.59	21.76 ± 0.50	***
A2780	23.37 ± 0.42	24.44 ± 0.17	***
IGROV1	22.81 ± 0.43	25.09 ± 0.07	***

Note: GLCM sampling offset was (10.0) and compare to WT and * *p* < 0.05, ** *p* < 0.01, and *** *p* < 0.001.
